# Dynamic Ultrasonographic Measurement of Inferior Joint Capsule Thickness in Patients with Unilateral Frozen Shoulder

**DOI:** 10.3390/diagnostics11050898

**Published:** 2021-05-18

**Authors:** Jun-Gyu Lee, Hyungsun Peo, Jang-Hyuk Cho, Chul-Hyun Cho, Don-Kyu Kim, Du-Hwan Kim

**Affiliations:** 1 Department of Physical Medicine and Rehabilitation, College of Medicine, Chung-Ang University, Seoul 06973, Korea; jglee412@caumc.or.kr (J.-G.L.); lovetheme3@caumc.or.kr (H.P.); donkim21@cau.ac.kr (D.-K.K.); 2 Department of Rehabilitation Medicine, Dongsan Medical Center, School of Medicine, Keimyung University, Daegu 42601, Korea; jacob.chojh@gmail.com; 3 Department of Orthopedic Surgery, Dongsan Medical Center, School of Medicine, Keimyung University, Daegu 42601, Korea; oscho5362@dsmc.or.kr

**Keywords:** frozen shoulder, ultrasound, inferior joint capsule

## Abstract

The diagnostic value of ultrasonography (US) for frozen shoulder (FS) is not well established. This study aimed to assess the diagnostic value of US measurement of inferior joint capsule (IJC) thickness and evaluate changes in the thickness of the IJC by US depending on arm position. A total of 71 patients with clinically diagnosed unilateral FS who underwent bilateral US measurement of the IJC were enrolled in this study. The US measurement of the IJC was performed with a linear transducer positioned around the anterior axillary line with the shoulder 40° abducted and with neutral rotation of the glenohumeral joint (neutral position). We also measured the IJC thickness in the externally rotated and internally rotated positions with the shoulder 40° abducted. In the neutral position, as well as in the internally rotated and externally rotated positions, the thickness of the IJC on US was significantly higher in the affected shoulder than that in the unaffected shoulder (all *p* < 0.001). On both the affected and unaffected sides, the US thickness of the IJC in the neutral position was significantly higher than that in the externally rotated position (*p* < 0.001), but lower than that in the internally rotated position (*p* < 0.001). Regarding IJC thickness in the neutral position, a 3.2-mm cutoff value yielded the highest diagnostic accuracy for FS, with a sensitivity and specificity of 73.2% and 77.5%, respectively. The area under the curve for IJC thickness was 0.824 (95% confidence interval, 0.76–0.89). US measurement of the IJC in the neutral position yielded good diagnostic accuracy for FS. Because IJC thickness is affected by arm rotation, it is important to measure the IJC thickness in a standardized posture to ensure diagnostic value.

## 1. Introduction

Frozen shoulder (FS) is a common shoulder disease characterized by pain and limited range of motion (ROM) [1]. FS is understood as a series of pathological processes in which the synovium of the glenohumeral joint is initially inflamed by unknown triggering factors and then gradually replaced by fibrosis; the joint then recovers naturally through an unknown mechanism [2,3,4,5,6]. It is not clear whether the lesion in FS spans the entire synovium of the glenohumeral joint or whether only specific areas are involved. However, previous studies using magnetic resonance imaging (MRI) and arthroscopic findings have consistently revealed that the inferior joint capsule (IJC) is a major site for lesions in FS [7,8,9,10,11,12].

There are two issues with the IJC in the FS. First, measurement of IJC thickness is known to be helpful in the differential diagnosis of FS [9,10]. FS is diagnosed based on the clinical findings of negative plain radiography, movement-associated pain, and restriction of ROM. Other stiff-shoulder conditions, such as rotator cuff tear, calcific tendinitis, and inflammatory arthropathy, can mimic FS. Increased thickness of the IJC on MRI or ultrasonography (US) is suggestive of synovitis representing primary or secondary FS [7,13,14,15,16]. According to the last clinical indications by the European Society of Musculoskeletal Radiology, US is not the first-choice technique for FS and should be considered if other imaging modalities are not appropriate [17]. However, a consensus for standardized methods measuring the thickness of the IJC on MRI or US is lacking. The second issue is the direction in which the IJC primarily affects the limited ROM of the FS [18,19,20]. This issue is related to the controversy regarding the extent of capsular release in the treatment of refractory FS and any rehabilitation strategy.

Measurement of IJC thickness can be performed using either MRI or US. US has advantages in that it is cheaper than MRI and allows bilateral comparison and dynamic testing. A few recent studies demonstrated heterogeneous results for US measurement of IJC, which might be related to non-standardized methods [15,16,21].

We aimed to assess the diagnostic value of US measurement of IJC thickness and evaluate changes in the thickness of the IJC on US depending on arm position and clarify the direction in which the IJC restricts the movement of the glenohumeral joint. We hypothesized that IJC thickness is affected by internal and external rotation of the glenohumeral joint.

## 2. Methods

### 2.1. Subjects

All patients who had a clinical diagnosis of unilateral FS and visited a single tertiary musculoskeletal US clinic between 2017 and 2019 to undergo US measurement of IJC thickness were eligible for inclusion in this study. The clinical diagnosis of FS was made by a single shoulder surgeon based on the patient’s history, physical examination findings, and plain radiography results. The clinical diagnosis of FS met the following criteria: (1) age ≥ 20 years, (2) shoulder pain with a limitation of passive motion greater than 30° in two or more planes of movement, and (3) normal plain radiography. Patients with bilateral FS, FS secondary to rheumatic disease, previous infectious arthritis around the shoulder, history of high-energy trauma, previous surgeries of the shoulder or adjacent regions, concomitant cervical radiculopathy, suspected rotator cuff disease, osteoarthritis, and insufficient medical records for clinical scores or past medical history were excluded. A total of 71 patients were enrolled in this study. This study was conducted according to the guidelines of the Declaration of Helsinki and approved by the Institutional Review Board of Dongsan Medical Center (IRB No: 2020-10-028, approved on 16 October 2020).

### 2.2. Us Protocol and Arm Positions

**Figure 1:**
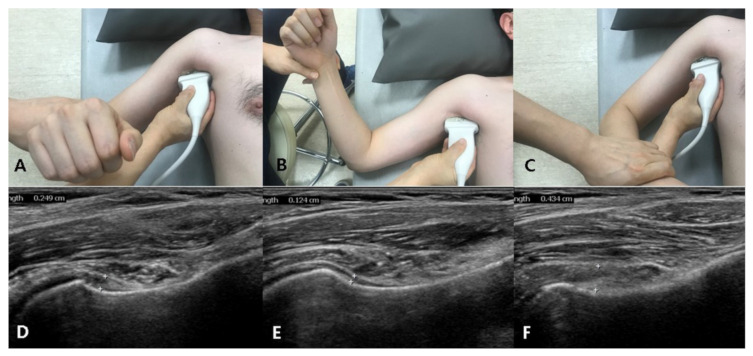
Arm positions and ultrasonography (US) measurement of inferior joint capsule (IJC) thickness. (**A**) Neutral position. (**B**) Externally rotated position. (**C**) Internally rotated position. (**D**–**F**) US measurement of IJC thickness according to arm position.

**Figure 2:**
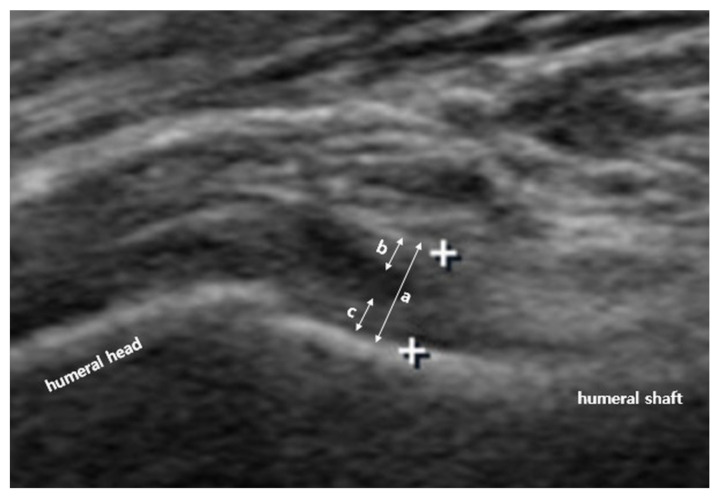
Ultrasonographic measurement of inferior joint capsule (IJC) thickness. IJC thickness was defined as the distance from the cortex of the humerus to the outer margin of the capsule at the widest portion of the capsule. IJC thickness (**a**) includes both the glenoid side (**b**) and humeral side (**c**).

US investigation of the IJC was performed by a single physiatrist with more than 15 years of experience in musculoskeletal US. A linear 5–12-MHz probe (HD15 ultrasound system; Philips, Amsterdam, The Netherlands) was used. Based on a previously reported protocol, to measure the IJC thickness in the neutral position, the patient was laid in a supine position with the shoulder abducted 40° and elbow flexed 90°; the transducer was placed around the anterior axillary line to visualize the cortex of the humerus (Figure 1). To measure the IJC thickness in the externally rotated and internally rotated position, the patients maximally rotated their upper arm with their palms facing the sky or ground, respectively, with the shoulder abducted 40° (Figure 1). A still image that best showed the IJC near the anatomic neck of the humerus was selected and saved, and the IJC thickness was measured using the caliper of the ultrasound machine. IJC thickness was defined as the distance from the cortex of the humerus to the outer margin of the capsule at the widest portion of the capsule (Figure 2). Thus, the IJC thickness includes both the humeral and glenoid sides that make up the entire IJC (Figure 2). These parameters were measured on both the affected and unaffected sides.

### 2.3. Range of Motion Assessment

Passive ROM was measured with the patient in a sitting position by a single shoulder surgeon blinded to the patient’s US findings. The forward flexion, abduction, and external rotation angles in a neutral position were measured using a steel goniometer. Scapular rotation movement was allowed for the measurement of forward flexion and abduction. To measure the angle of internal rotation, the level of the spine where the tip of the thumb reached was recorded using a scratch test. The measured vertebral level was then converted into consecutive numbers from 1 to 20 as follows: T1–T12, 1–12; L1–L5, 13–17; sacrum, 18; buttock, 19; trochanter, 20.

### 2.4. Statistical Analysis

IBM Statistical Package for Social Sciences (SPSS) version 21 software (Armonk, New York, NY, USA) was used for data analysis. The Wilcoxon signed-rank tests were used to compare the IJC thickness on US between the affected and unaffected sides. The Mann–Whitney test was used to compare the IJC thickness between the two different positions. The correlation between the normalized dynamic change in IJC thickness between two different positions and the ROM was assessed using Pearson’s correlation coefficient. The normalized dynamic change in IJC thickness was defined as the difference in the IJC thickness between that in the neutral position and externally rotated position or internally rotated position, which was normalized relative to the IJC thickness at the neutral position. A receiver operating characteristic curve and the area under the curve (AUC) for IJC thickness on US in the neutral position were calculated. The sensitivity, specificity, and Youden index were calculated for 3.0-, 3.2-, and 3.5-mm cutoff values to establish the diagnostic accuracy for the diagnosis of FS. All statistical tests were conducted at the two-sided 5% significance level, and all reported *p*-values are two-sided. Statistical significance was set at *p* < 0.05.

## 3. Results

### 3.1. Characteristics of the Patients

The clinical characteristics of the patients are summarized in Table 1. The mean age was 58.2 ± 9.9 years; 45 patients (63.3%) were female, and 26 patients (36.7%) were male. The mean duration of symptoms at the first visit was 6.7 ± 3.2 months (range, 3–14 months). Eight patients (11.3%) had type II diabetes mellitus.

### 3.2. Comparison of the IJC Thickness on Us between the Affected and Unaffected Shoulders

**Figure 3:**
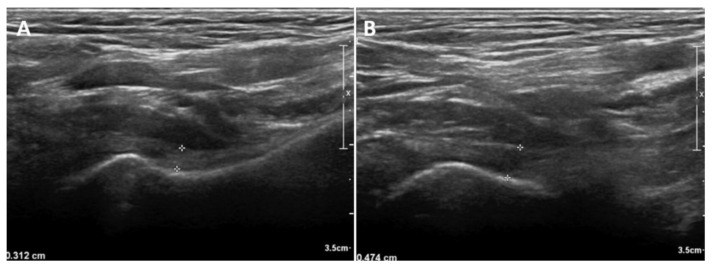
An illustrative case with unilateral frozen shoulder demonstrating the difference in inferior joint capsule (IJC) thickness in neutral position. (**A**) IJC thickness of the unaffected side. (**B**) IJC thickness of the affected side.

**Figure 4:**
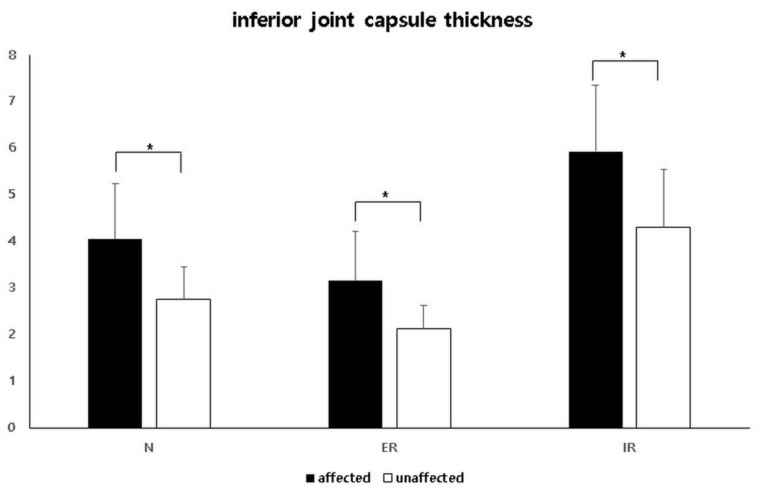
Comparison of the inferior joint capsule thickness between the affected and unaffected shoulders in three different positions. N, neutral position; ER, externally rotated position; IR, internally rotated position. * Statistically significant.

In neutral position, the IJC thickness on US was significantly higher in the affected shoulder (mean, 4.04 mm; standard deviation [SD], 1.19; 95% confidence interval [CI], 3.76–4.31) than that in the unaffected shoulder (mean, 2.76 mm; SD, 0.70; 95% CI, 2.59–2.92) (*p* < 0.001). An illustrative case is shown in Figure 3. In the externally rotated position, the IJC thickness on US was significantly higher in the affected shoulder (mean, 3.15 mm; SD, 1.07; 95% CI, 2.90–3.40) than in the unaffected shoulder (mean, 2.12 mm; SD, 0.50; 95% CI 2.00–2.24) (*p* < 0.001). In the internally rotated position, the IJC thickness on US was significantly higher in the affected shoulder (mean, 5.92 mm; SD, 1.43; 95% CI, 5.59–6.25) than in the unaffected shoulder (mean, 4.30 mm; SD, 1.24; 95% CI, 4.01–4.59) (*p* < 0.001) (Table 2, Figure 4).

### 3.3. Comparison of the IJC Thickness on Us Depending on Arm Position

**Figure 5:**
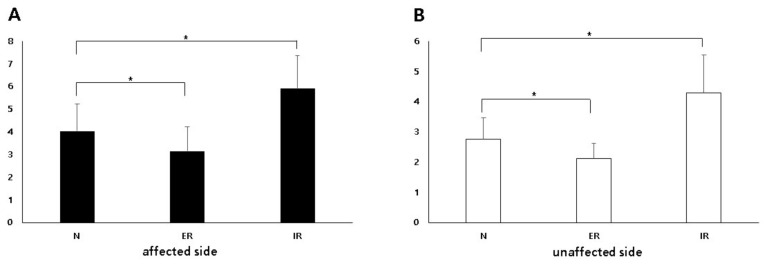
Comparison of the inferior joint capsule thickness between three different positions on both the affected (**A**) and unaffected (**B**) sides. * Statistically significant.

On both the affected and unaffected sides, the IJC thickness on US in the neutral position was significantly higher than that in the externally rotated position, but less than that in the internally rotated position (Table 2, Figure 5).

### 3.4. Diagnostic Cutoff Value for IJC Thickness on Us in the Neutral Position

**Figure 6:**
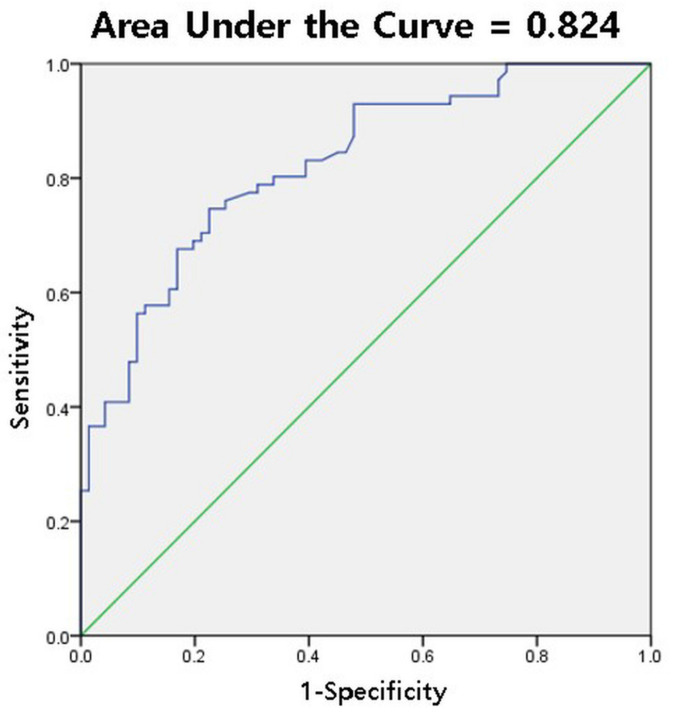
Receiver operating characteristic curve and area under curve (AUC) of inferior joint capsule thickness on ultrasonography in the neutral position for the diagnosis of frozen shoulder (AUC = 0.824).

For the IJC thickness, a 3.2-mm cutoff value yielded the highest diagnostic accuracy for FS, with a sensitivity and specificity of 73.2% and 77.5%, respectively (Youden index = 0.507). The AUC for IJC thickness was 0.824 (95% CI, 0.76–0.89) (Table 3, Figure 6).

### 3.5. Correlation between the Dynamic Change in IJC Thickness US and ROM

We evaluated the correlation between the normalized dynamic change in the IJC thickness by US and ROM measurement. The normalized dynamic change in IJC thickness between the neutral position and external rotated position significantly correlated with only the forward flexion angle, but the others did not significantly correlate with any ROM (Table 4).

## 4. Discussion

This study assessed the diagnostic value of IJC thickness measured by US for the diagnosis of FS and evaluated the changes in the thickness of the IJC depending on arm position. A cutoff value of 3.2 mm for IJC thickness on US yielded good diagnostic accuracy for FS with a sensitivity and specificity of 73.2% and 77.5%, respectively. The IJC thickness was dependent on arm position. The IJC thickness tended to be lower as the glenohumeral joint was externally rotated.

US is known to be comparable to MRI in its diagnostic accuracy for rotator cuff lesions; however, its usefulness in the diagnosis of FS remains inconclusive. Recently, attempts have been made to use US to diagnose FS [7,15,16,21,22,23,24,25]. The thickness of the coracohumeral ligament, vascularity of the rotator interval, and thickness of the IJC on US were used for the diagnosis of FS [15,16,24,25,26]. Among these three variables, the results for IJC thickness were relatively consistent, demonstrating that IJC thickness of the affected side was significantly greater than that of the unaffected side [15,16,21]. However, a diagnostic cutoff value for IJC thickness on US has not been established. Cheng et al. reported that using a 3.5-mm cutoff value, the sensitivity and specificity were 66.7% and 93.3%, respectively [22]. Our results showed that a cutoff value of 3.2 mm for IJC thickness on US yielded a sensitivity and specificity of 73.2% and 77.5% for FS diagnosis. The cutoff value (3.2 or 3.5 mm) for IJC thickness on US, including both the humeral and glenoid side capsule, in the diagnosis of FS would be somewhat different from that on MRI (only including the humeral side, 3.0–5.0 mm) [11,12,14,15,27]. Although the cutoff values were heterogeneous because of the different regions of interest when measuring the IJC on MRI, the cutoff value on US would be lower than that on MRI because the joint capsule is more tense while the patient’s shoulder is abducted.

The results of US measurements of IJC thickness have been heterogeneous. Sernik et al. reported that IJC thickness greater than 2.0 mm correlated with MRI signs of FS [21]. In that study, the gold standard for the diagnosis of FS was MRI, not clinical findings, such as ROM limitation. In addition, US examination was performed while the shoulder position was 90° abducted and 90° externally rotated. We believe that this position might be impossible in many patients with FS because of ROM limitations. It seems that a large proportion of patients without true FS might have been included in that study. Michelin et al. also demonstrated that the mean thickness (4.0 mm) of the affected side in patients with FS was significantly greater than that on the asymptomatic side (1.3 mm) [15]. In that study, US examination was conducted with the shoulders maximally abducted. Predictably, the unaffected side would be abducted to almost 90°, and few patients would be able to abduct their affected shoulders to 90°.

We believe that it is necessary to standardize the US measurement method for IJC thickness for it to have diagnostic value in diagnosing FS. Since it is difficult for patients with FS to abduct the shoulder 90° in the supine position, we decided to measure the IJC thickness bilaterally with shoulder abduction at 40° in this study. When measuring IJC thickness in the clinical field, our authors found that the IJC thickness was dependent on rotation as well as shoulder abduction. Our results revealed that the IJC thickness on US in the neutral position was significantly higher than that in the externally rotated position. The IJC measured using US in this study corresponded strictly to the anterior portion of the inferior capsule. Our results suggest that the anterior IJC might be stretched during external rotation at 40° abduction.

Although FS is a self-limiting disease, capsular release is sometimes performed for refractory FS. The golden rule in the treatment of refractory FS is the release of the coracohumeral ligament (CHL) and thickened capsule. However, “how much release is needed” has often been debated. Hagiwara et al. reported that all segments of the joint capsule affected ROM in all directions, suggesting that whole-joint capsular release is necessary to achieve sufficient ROM gain in patients with FS [20]. Chen et al. reported that there was no significant difference between an anterior inferior release and an extended posterior “270° release” [28]. Previous cadaveric studies revealed that the CHL restrained external rotation below 60° abduction and the anterior inferior glenohumeral ligament was tense in abduction, extension, and external rotation [18,19]. Our study demonstrated the role of the anterior IJC in restraining external rotation in patients with FS. Our study suggests that the release of the anterior IJC and CHL could at least be considered in patients with severe restriction of external rotation. Because we did not perform a biomechanical study and the patients did not undergo surgery that suggest an improvement of external rotation after the release of the anterior IJC, this conceptual explanation would not be actually guaranteed, and further investigation is necessary in future studies.

This study has some limitations. First, our study was retrospectively designed, so there is a potential selection bias. Second, we did not compare the FS group with groups with other shoulder pathologies as controls because other causes of shoulder pain may also demonstrate increased IJC thickness on US. Further studies are needed to elucidate whether the increased IJC thickness on US is pathognomonic for FS. Third, we did not assess the reliability of US measurement of IJC thickness in patients with FS, although a previous study revealed good reliability in a healthy population [29]. Lastly, we tried to maintain a standardized position of shoulder abduction; however, there might have been some movements because the patient’s shoulder was not completely fixed.

## 5. Conclusions

The IJC thickness on US in the neutral position was significantly higher than that in the externally rotated position. With shoulder abduction at 40° and neutral rotation, a cutoff value of 3.2 mm for IJC thickness on US yielded a sensitivity and specificity of 73.2% and 77.5%, respectively. Because the IJC thickness on US was dependent on arm position, it is important to measure IJC thickness in a standardized posture to secure diagnostic value.

## Figures and Tables

**Table 1:** Patient characteristics.

Characteristic	Value
Number of patients	71
Mean age, years	58.2 ± 9.9
Sex: male, female	26, 45
Duration of symptoms, months (range)	6.7 ± 3.2 (3–14)
Number of diabetic patients	8
Forward flexion (°)	115.6 ± 18.4
Abduction (°)	96.8 ± 20.2
External rotation (°)	40.1 ± 11.3
Internal rotation score	16.7 ± 2.1

Values are presented as mean ± standard deviation.

**Table 2:** Comparison of the IJC thickness on US between the affected and unaffected shoulders and between three different positions.

	N	ER	IR	*p*-Value (N vs. ER)	*p*-Value (N vs. IR)	*p*-Value (Affected vs. Unaffected)
Affected side	4.04 ± 1.19	3.15 ± 1.07	5.92 ± 1.43	<0.001	<0.001	N: <0.001, ER: <0.001, IR: <0.001
Unaffected side	2.76 ± 0.70	2.12 ± 0.50	4.30 ± 1.24	<0.001	<0.001	

N, neutral position; ER, externally rotated position; IR, internally rotated position.

**Table 3:** Diagnostic accuracy according to cutoff value of inferior joint capsule (IJC) thickness on ultrasonography.

IJC Thickness (mm)	Sensitivity	Specificity	Youden Index
3.0	77.5%	69.0%	0.465
3.2	73.2%	77.5%	0.507
3.5	66.2%	83.1%	0.493

**Table 4:** Correlation between normalized dynamic changes in inferior joint capsule (IJC) thickness and range of motion.

	ΔIJC_ER_		ΔIJC_IR_	
	r	*p*	r	*p*
Forward flexion	0.259	0.020^*^	0.023	0.849
Abduction	0.116	0.336	0.056	0.644
External rotation	0.165	0.168	0.150	0.212
Internal rotation	−0.114	0.346	0.135	0.262

ΔIJC_ER_: (IJC at neutral position—IJC at externally rotated position)/IJC at neutral position. ΔIJC_IR_: (IJC at internally rotated position—IJC at neutral position)/IJC at neutral position. * Statistically significant.

## Data Availability

The data presented in this study are available on request from the corresponding author.
